# Synthesis and crystal structure of poly[(2,6-di­methyl­py­ra­zine-κ*N*^4^)(μ_3_-thiocyanato-κ^3^*N*:*S*:*S*)copper(I)]

**DOI:** 10.1107/S2056989026001866

**Published:** 2026-02-24

**Authors:** Christian Näther

**Affiliations:** a Institut für Anorganische Chemie, Universität Kiel, Max-Eyth.-Str. 2, 24118 Kiel, Germany; University of Aberdeen, United Kingdom

**Keywords:** crystal structure, coordination polymer, copper(I) thio­cyanate, 2,6-di­methyl­pyrazine

## Abstract

Crystals of the title compound, [Cu(NCS)(C_6_H_8_N_2_)]_*n*_ (C_6_H_8_N_2_ = 2,6-di­methyl­pyrazine), were prepared by the reaction of CuNCS and 2,6-di­methyl­pyrazine in aceto­nitrile. The asymmetric unit consists of one Cu^I^ cation, one thio­cyanate anion and one 2,6-di­methyl­pyrazine ligand with all atoms lying on general positions. The copper cations are tetra­hedrally coordinated by two S- and one N-bonded thio­cyanate anions and one 2,6-di­methyl­pyrazine ligand, which s coordinated to the metal center with the N atom that is not adjacent to the methyl groups. The copper cations are linked by the μ-1,3,3 (*N*,*S*,*S*) bridging thio­cyanate anions into layers that lie parallel to the *ac* plane. The layers are stacked perpendicular to the *b*-axis direction and are separated by the 2,6-di­methyl­pyrazine ligands. The title crystal structure is compared with those of related CuNCS compounds with isomeric di­methyl­pyrazine ligands.

## Abstract 2

In the title compound, the copper(I) cations are tetra­hedrally coordinated and linked into layers by μ-1,3,3(*N*,*S*,*S*) bridging thio­cyanate anions.

## 1. Chemical context

Coordination compounds based on copper(I) halides with chloride, bromide and iodide anions and N-donor coligands show an extremely large structural variability (*e.g*., Kromp & Sheldrick, 1999 ▸; Näther *et al.*, 2001 ▸, 2002 ▸; Li *et al.*, 2005 ▸; Peng *et al.*, 2010 ▸). They usually consist of a variety of Cu*X* substructures such as monomeric and dimeric units, chains or layers that can be further linked if bridging ligands are used. This might also be one reason why several polymorphs or isomers are reported (Näther & Jess, 2003 ▸; Park *et al.*, 2012 ▸; Peng *et al.*, 2010 ▸; Näther *et al.*, 2003 ▸). For many of these compounds, a different ratio between the copper(I) halide and the N-donor coligands is observed and thermal treatment of the coligand-rich compounds usually leads to the transformation into coligand-deficient compounds that show more condensed Cu*X* substructures (Näther & Jess, 2001 ▸, 2002 ▸).

Such coordination compounds can also be prepared with copper(I) pseudohalides such as cyanide, azide or thio­cyanate anions and many of them are reported in the literature because of their luminescence properties (Chesnut *et al.*, 1999 ▸; Lemos *et al.*, 2001 ▸; Starosta *et al.*, 2012 ▸; Nitsch *et al.*, 2015 ▸). As is the case for the copper(I) halide coordination compounds, they also show typical Cu*X* substructures (*X* = pseudohalide), which, especially for cyanides, are very often more complicated than those in copper(I) halides. The different Cu*X* substructures can further be connected into more condensed networks if bridging coligands such as, for example, pyrazine derivatives are used. If a database search is limited to the isomeric di­methyl­pyrazine ligands and copper(I), a number of compounds with cyanide, azide and thio­cyanate anions are reported in the CSD (Version 5.43, 2025; Groom *et al.*, 2016 ▸) using CONQUEST (Bruno *et al.*, 2002 ▸).

With azide anions, no copper(I) compounds with 2,3-di­methyl­pyrazine are reported but one three-dimensional compound with the composition Cu_2_(N_3_)_2_(2,5-di­methyl­pyrazine) is known that shows a complicated Cu–azide substructure, in which the azide anions act as μ-1,1,3 bridging ligands (Guang *et al.*, 2012 ▸). Furthermore, Cu_2_(N_3_)_2_(2,6-di­methyl­pyrazine) is also found (Fan *et al.*, 2015*a* ▸,*b* ▸).

Copper(I) compounds with cyanide anions are reported with all three isomers of di­methyl­pyrazine. These include Cu_3_(CN)_3_(2,3-di­methyl­pyrazine) (Greve & Näther, 2004 ▸), Cu_6_(CN)_6_(2,3-di­methyl­pyrazine) (Chesnut *et al.*, 2001 ▸) and Cu_2_(CN)_2_(2,5-di­methyl­pyrazine) (Chesnut *et al.*, 2001 ▸). Finally, two isomers of Cu_2_(CN)_2_(2,6-di­methyl­pyrazine) are reported. In one of them, the copper cations are linked by bridging cyanide anions into Cu_4_(CN)_4_ units that condense into layers by way of Cu_2_(CN)_2_ four-membered rings (Näther, 2025 ▸), whereas in the second modification a one-dimensional copper(I) cyanide network is found that consists of alternating twelve- and four-membered rings (Chesnut *et al.*, 2001 ▸).

With thio­cyanate anions and 2,5-di­methyl­pyrazine, a compound with the composition Cu_2_(NCS)_2_(2,5-di­methyl­pyrazine) is found, in which CuNCS layers are observed, that are linked by the 2,5-di­methyl­pyrazine ligands into a three-dimensional network (Näther *et al.*, 2003 ▸; Otieno *et al.*, 2003 ▸). A three-dimensional structure is also found for Cu_2_(NCS)_2_(2,3-di­methyl­pyrazine), even if the layer topology is different from that of the 2,5-di­methyl­pyrazine compound (Näther *et al.*, 2003 ▸). Compounds with a 1:1 ratio of copper(I) thio­cyanate and 2,6-di­methyl­pyrazine are unknown and we therefore tried to prepare such compounds by the reaction of CuNCS and 2,6-di­methyl­pyrazine. In the course of these investigations we obtained crystals of the title compound, (**I**), that were characterized by single-crystal X-ray diffraction.
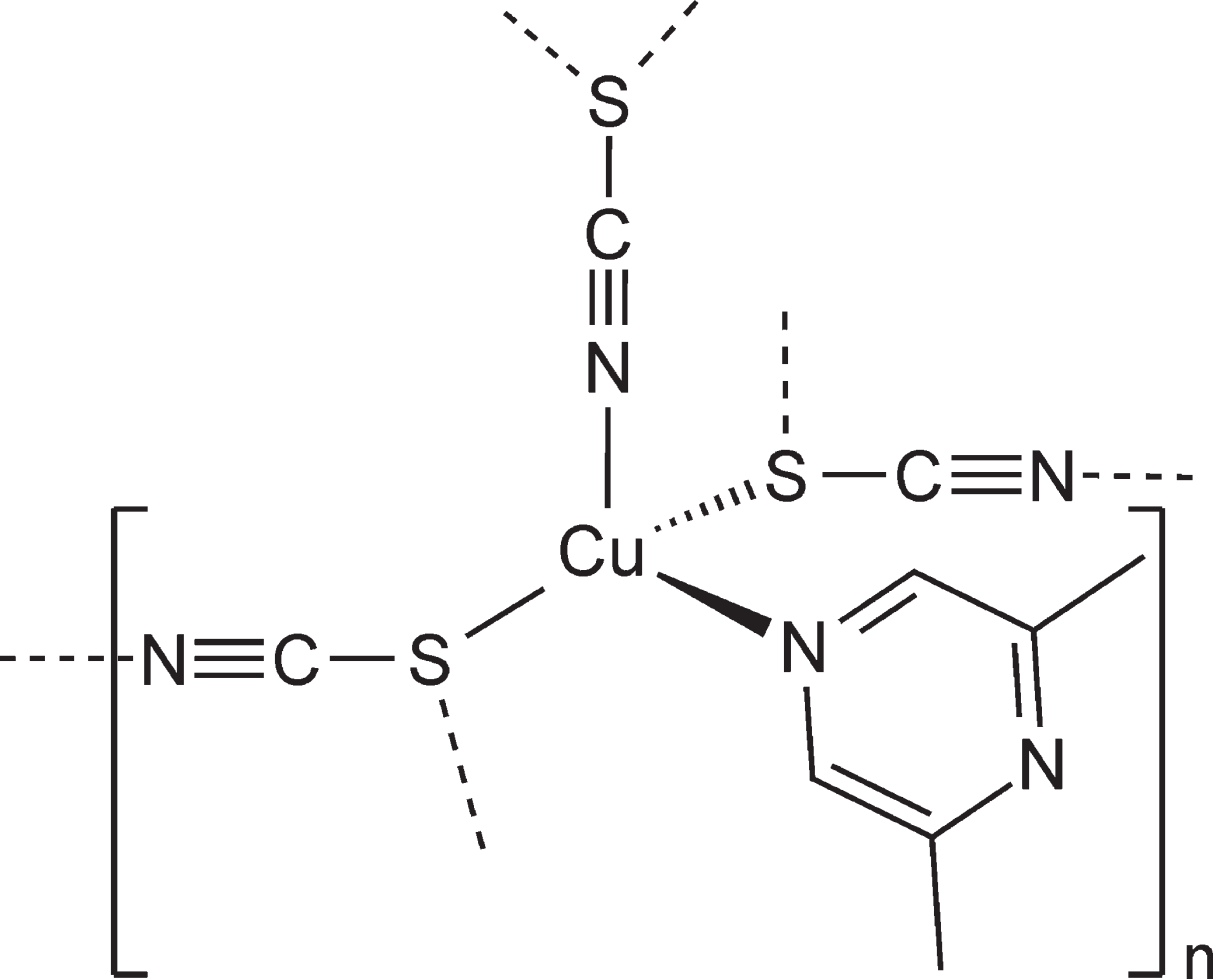


## 2. Structural commentary

**Figure 1:**
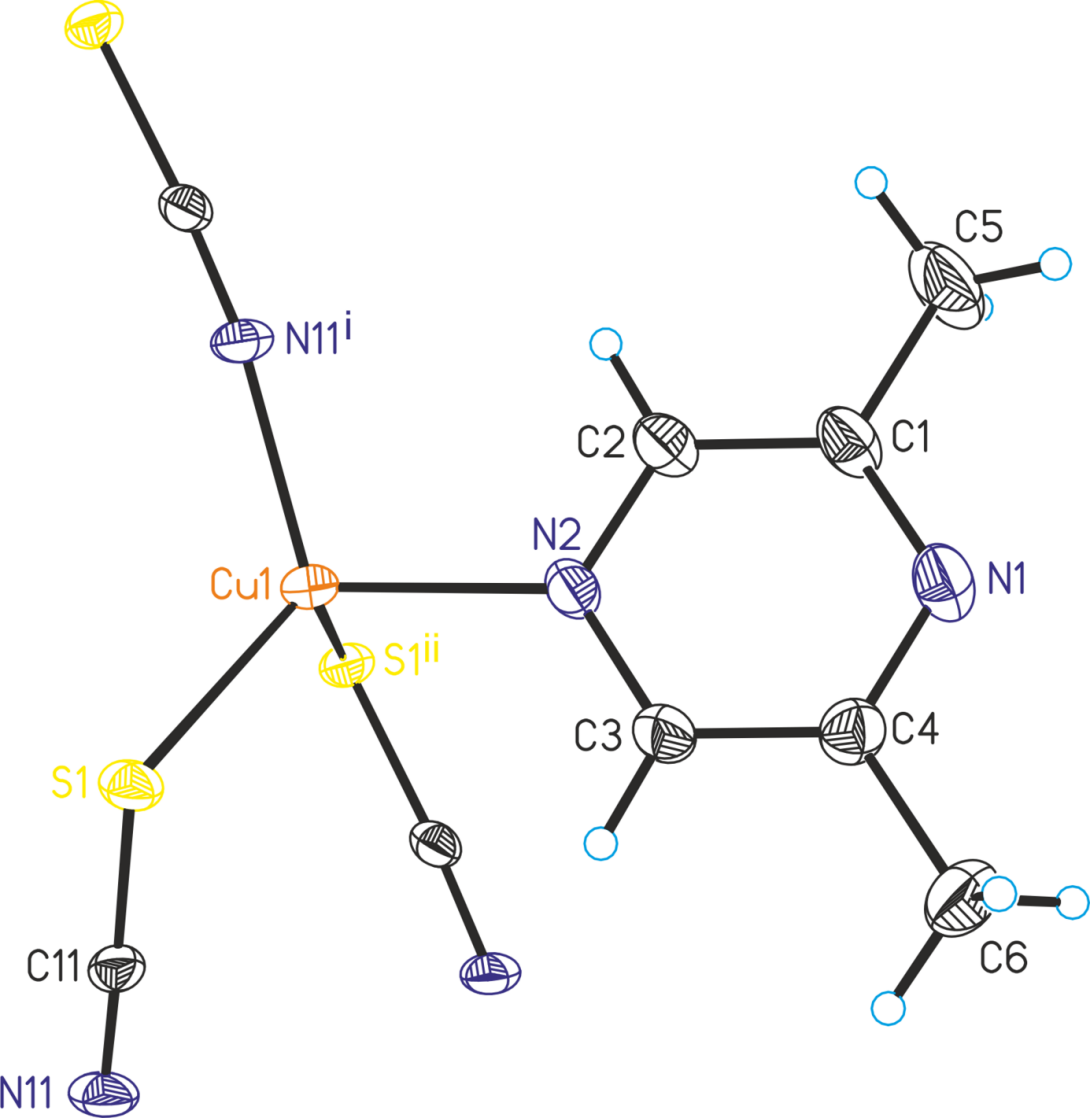
The asymmetric unit of (**I**) expanded to show the full metal coordination sphere with labeling of selected atoms and displacement ellipsoids drawn at the 50% probability level. Symmetry codes: (i) *x* − 1, −*y* + 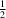, *z -* 1/2; (ii) *x*, −*y* + 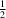, *z* − 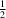.

The asymmetric unit of (**I**), Cu(NCS)(C_6_H_8_N_2_) (C_6_H_8_N_2_ = 2,6-di­methyl­pyrazine), consists of one copper(I) cation, one thio­cyanate anion and one 2,6-di­methyl­pyrazine ligand, with all of the atoms located in general positions (Fig. 1 ▸) in space group *P*2_1_/*c*. The metal cations are fourfold coordinated by one N- and two S-bonded thio­cyanate anions and one 2,6-di­methyl­pyrazine ligand. Because of steric repulsion between the metal cation and the methyl groups of the 2,6-di­methyl­pyrazine ligand, this ligand is only coordinated with the N atom that does not lie between the two methyl groups (Fig. 1 ▸). The two Cu—S bond lengths are only slightly different and the bond angles deviate from the ideal values, which shows that the tetra­hedra are slightly distorted (Table 1 ▸). As expected, the C—N—Cu angle is close to linearity, whereas the C—S—Cu angles roughly correspond to a tetra­hedral angle (Table 1 ▸).

**Figure 2:**
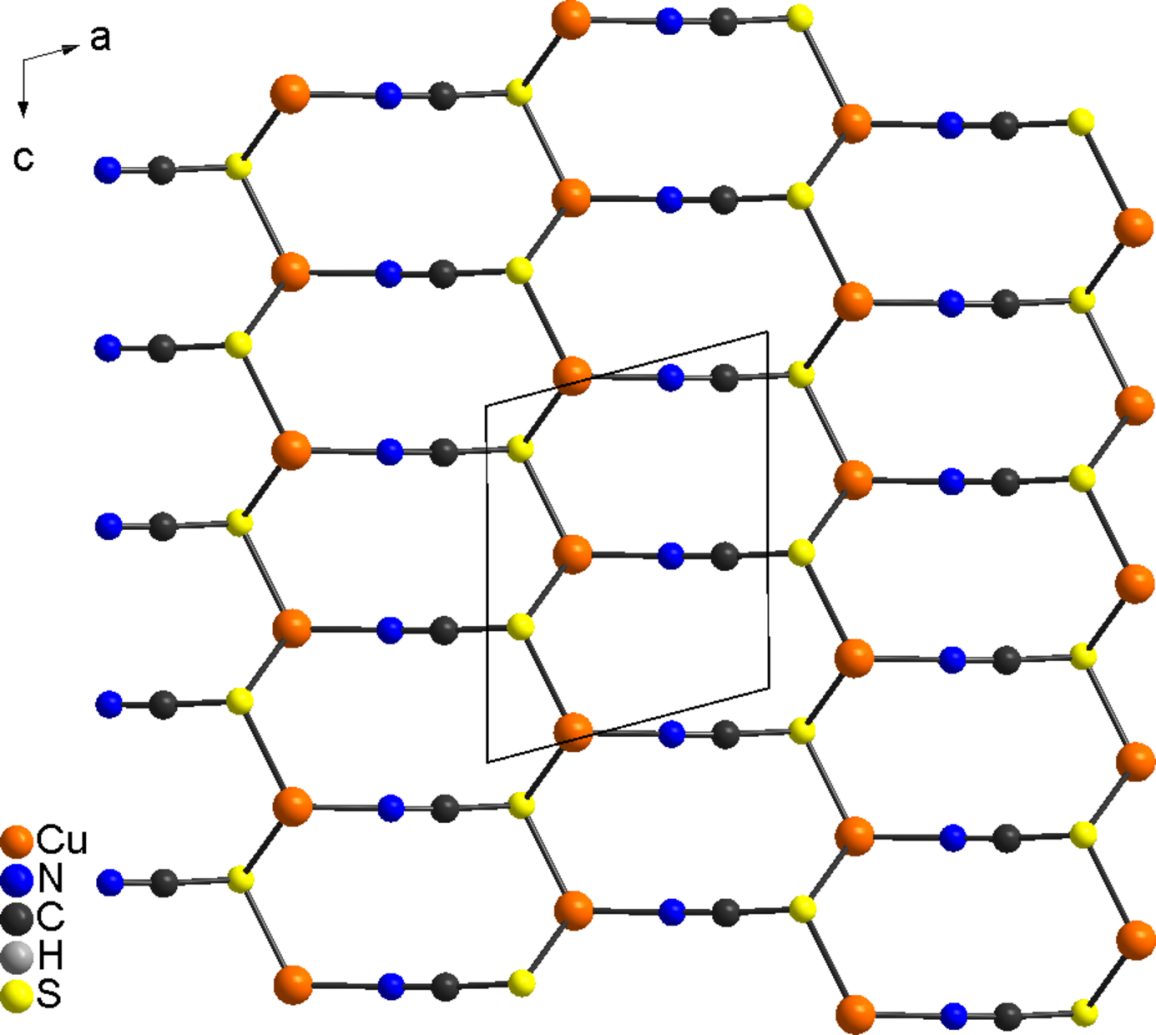
Crystal structure of (**I**) with a view onto the CuNCS layers along the crystallographic *b*-axis direction. The 2,6-di­methyl­pyrazine ligands are omitted for clarity.

**Figure 3:**
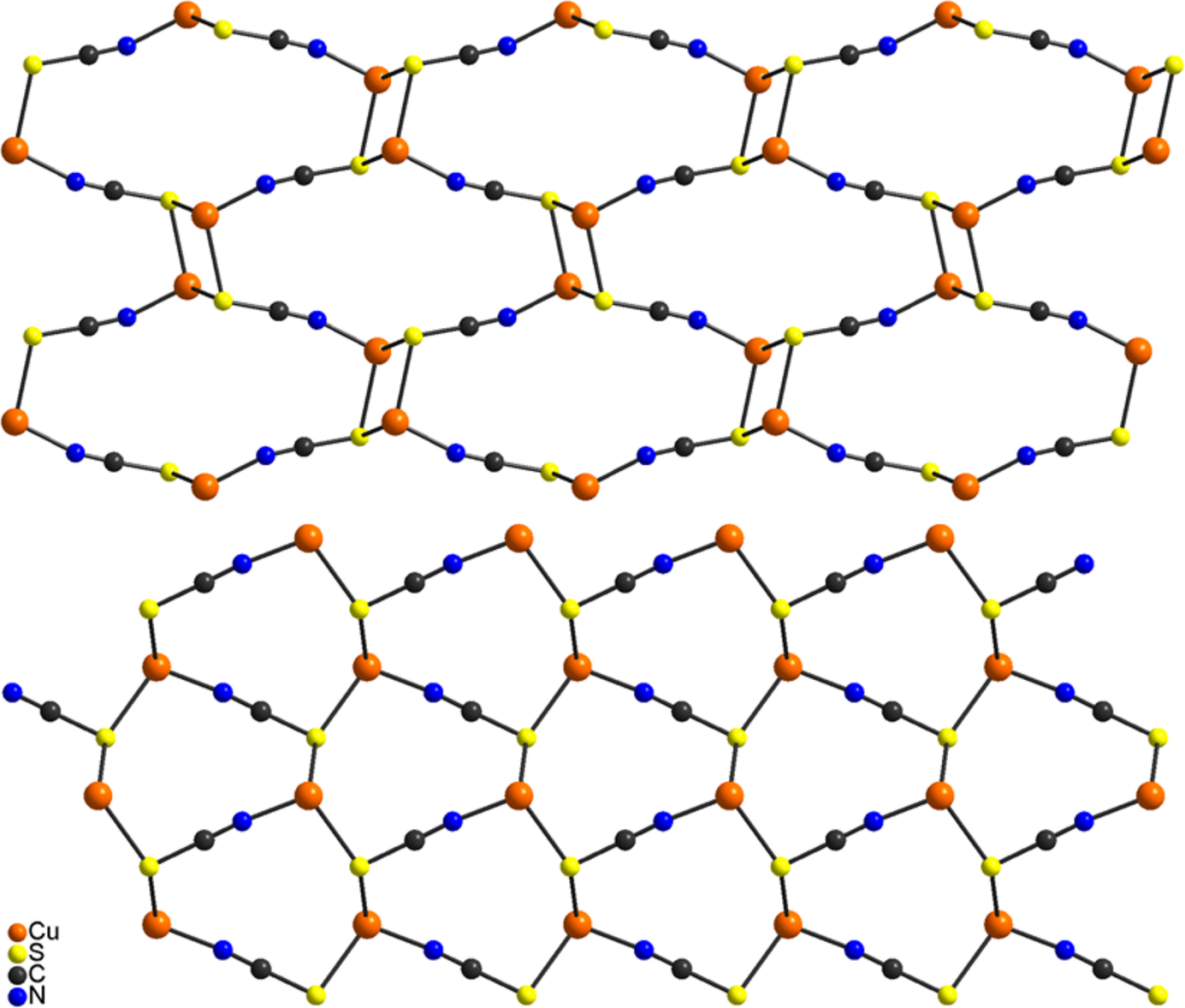
View of the CuNCS networks in Cu_2_(NCS)_2_(2,3-di­methyl­pyrazine) (top) and Cu_2_(NCS)_2_(2,5-di­methyl­pyrazine) (bottom) reported in the literature (Näther *et al.*, 2003 ▸; Otieno *et al.*, 2003 ▸).

In the extended structure, the copper(I) cations are connected by μ-1,1,3(*S*,*S*,*N*)-bridging thio­cyanate anions into ten-membered rings built up of three cations and three thio­cyanate anions, condensing into corrugated layers that lie parallel to the *ac* plane (Fig. 2 ▸). It is noted that this layer topology is completely different from that in Cu_2_(NCS)_2_(2,3-di­methyl­pyrazine) (Näther *et al.*, 2003 ▸), where tetra­nuclear units built up of four copper(I) cations and four thio­cyanate anions are observed, which condense into layers by way of Cu_2_S_2_ rings (Fig. 3 ▸: top). In contrast, in Cu_2_(NCS)_2_(2,5-di­methyl­pyrazine) (Näther *et al.*, 2003 ▸; Otieno *et al.*, 2003 ▸), ten-membered rings are also found but the orientation of the two thio­cyanate anions within these rings is reversed and the rings are therefore more distorted (Fig. 3 ▸: bottom).

Concerning the overall structural discussion, it must be kept in mind that in the 2,3- and 2,5-di­methyl­pyrazine compounds, the ratio between the CuNCS component and the di­methyl­pyrazine derivative is different, but this difference only originates from the fact that the coligand is only terminally coordinating in (**I**), whereas it act as a bridging ligand in the compounds with the two other isomers. In this context, it is noted that no CuNCS compounds with 2,3- or 2,5-di­methyl­pyrazine are reported, in which the ratio between CuNCS and coligand is identical to that in (**I**), which indicates that for these ligands a terminal coordination is not favored. In contrast, in (**I**), one of the coordinating N atoms of the 2,6-di­methyl­pyrazine ligand is shielded by the two neighbouring methyl groups, which means that a compound with a bridging coordination of the neutral coligand, similar to that in the thio­cyanate compounds with 2,3- and 2,5-di­methyl­pyrazine, might not exist.

## 3. Supra­molecular features

**Figure 4:**
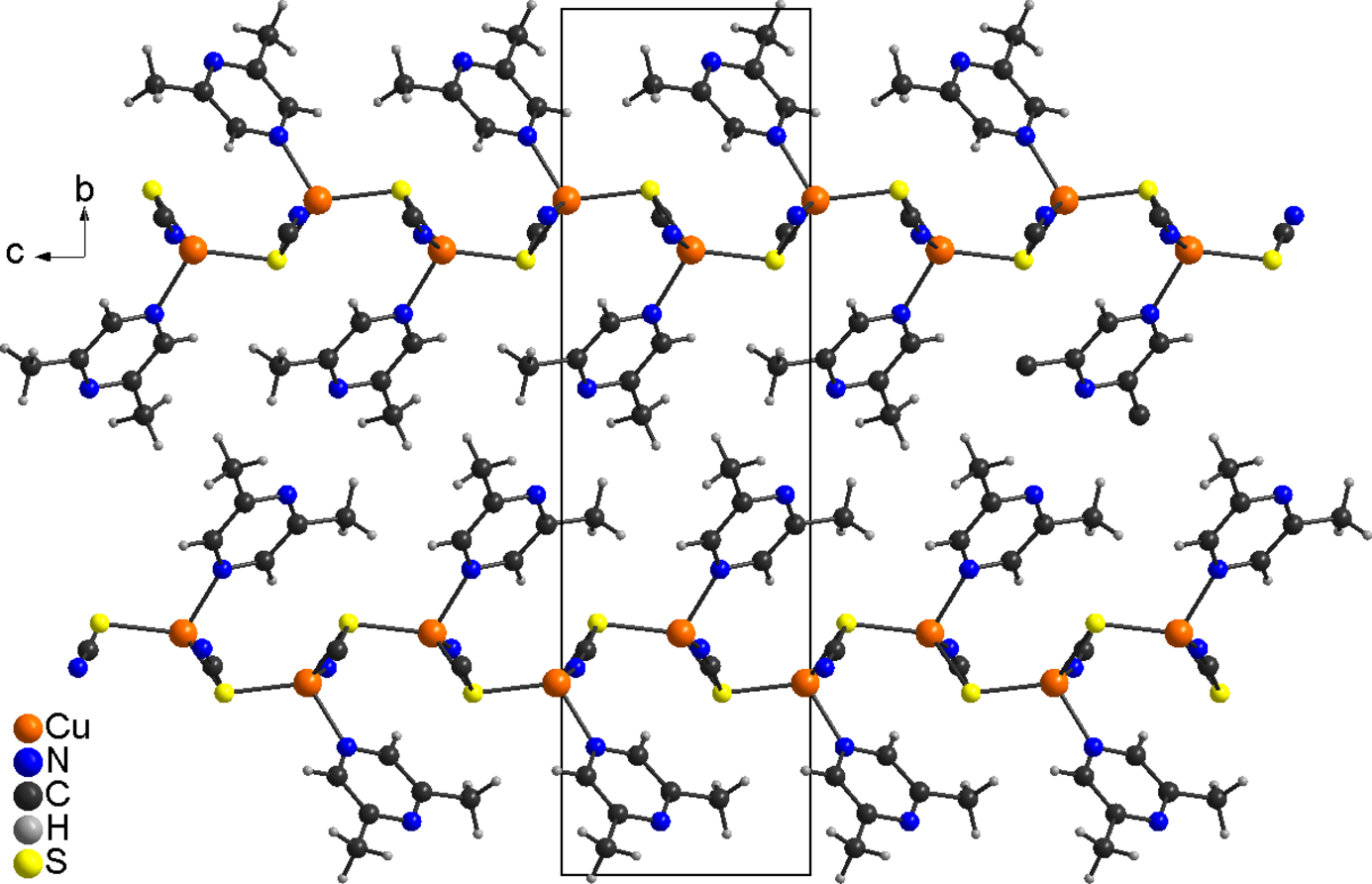
Crystal structure of (**I**) with a view along the crystallographic *a*-axis direction.

The layers in (**I**) are stacked perpendicular to the *b-*axis direction and are separated by the 2,6-di­methyl­pyrazine ligands (Fig. 4 ▸). The coligands of neighboring layers point towards each other, which means that the layers are only linked by van der Waals inter­actions. There are no directional inter­molecular inter­actions. This is completely different to the 2,3- and 2,5-di­methyl­pyrazine compounds Cu_2_(NCS)_2_(2,3-di­methyl­pyrazine) and Cu_2_(NCS)_2_(2,5-di­methyl­pyrazine) in which the layers are connected by bridging 2,3- and 2,5-di­methyl­pyrazine ligands into a three-dimensional network (Näther *et al.*, 2003 ▸; Otieno *et al.*, 2003 ▸). As mentioned above, this might be traced back to the fact that in 2,3- and 2,5-di­methyl­pyrazine, only one methyl group is adjacent to the coordinating N atom, whereas in 2,6-di­methyl­pyrazine the two methyl groups effectively shield one of the N atoms, which makes metal coordination much more difficult.

## 4. Database survey

As mentioned above, with 2,6-di­methyl­pyrazine and copper(I) thio­cyanate no compounds are reported but there is one mixed copper(I/II) pseudohalide compound with the composition [Cu_8_^I^Cu_2_^II^(CN)_4_(NCS)_8_(2,6-di­methyl­pyrazine)_7_] that shows a three-dimensional coordination network (Jess & Näther, 2006 ▸).

With copper(I) halides, two compounds with 2,6-di­methyl­pyrazine are known. This includes Cu_2_Cl_2_(2,6-di­methyl­pyrazine), in which the copper cations are tetra­hedrally coordinated by three chloride anions and one 2,6-di­methyl­pyrazine ligand and are linked by μ-1,1 bridging chloride anions into double chains that are further connected into layers by bridging 2,6-di­methyl­pyrazine ligands (Fan *et al.*, 2015 ▸ ▸). CuI(2,6-di­methyl­pyrazine) shows a structure similar to that of Cu_2_Cl_2_(2,6-di­methyl­pyrazine) mentioned above, but in this compound, the 2,6-di­methyl­pyrazine ligand is only terminally coordinated (Kitada & Ishida, 2014 ▸; Zhang *et al.*, 2014 ▸).

Finally, it is noted that with divalent copper(II) cations, two different polymorphs with the composition CuBr_2_(2,6-di­methyl­pyrazine) are reported, in which the copper cations are linked into chains by bridging 2,6-di­methyl­pyrazine ligands (Ding *et al.*, 2021 ▸).

## 5. Synthesis and crystallization

Copper(I) thio­cyanate and 2,6-di­methyl­pyrazine were purchased from Sigma-Aldrich: 1.000 mmol (121.6 mg) of copper(I) thio­cyanate and 1.000 mmol (108.1 mg) of 2,6-di­methyl­pyrazine were reacted in 3 ml of aceto­nitrile. Within 3 d, colourless blocks of (**I**) suitable for single crystal X-ray diffraction were obtained.

## 6. Refinement

Crystal data, data collection and structure refinement details are summarized in Table 2 ▸. The C—H hydrogen atoms were positioned with idealized geometry (methyl H atoms allowed to rotate but not to tip) and were refined isotropically with *U*_iso_(H) = 1.2*U*_eq_(C) (1.5 for methyl H atoms).

## Supplementary Material

Crystal structure: contains datablock(s) I. DOI: 10.1107/S2056989026001866/hb8197sup1.cif

Structure factors: contains datablock(s) I. DOI: 10.1107/S2056989026001866/hb8197Isup2.hkl

CCDC reference: 2532015

Additional supporting information:  crystallographic information; 3D view; checkCIF report

## Figures and Tables

**Table 1:** Selected geometric parameters (Å, °)

Cu1—N11^i^	1.963 (2)	Cu1—S1	2.3238 (8)
Cu1—N2	2.094 (2)	Cu1—S1^ii^	2.3809 (8)
			
N11^i^—Cu1—N2	105.53 (10)	S1—Cu1—S1^ii^	114.74 (3)
N11^i^—Cu1—S1	118.22 (7)	C11—S1—Cu1	107.96 (9)
N2—Cu1—S1	110.30 (7)	C11—S1—Cu1^iii^	96.46 (10)
N11^i^—Cu1—S1^ii^	105.40 (8)	Cu1—S1—Cu1^iii^	105.06 (3)
N2—Cu1—S1^ii^	100.90 (7)	C11—N11—Cu1^iv^	168.0 (3)

Symmetry codes: (i) 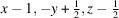; (ii) 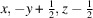; (iii) 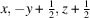; (iv) 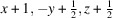.

**Table 2:** Experimental details

Crystal data	
Chemical formula	[Cu(NCS)(C_6_H_8_N_2_)]
*M*_r_	229.76
Crystal system, space group	Monoclinic, *P*2_1_/*c*
Temperature (K)	200
*a*, *b*, *c* (Å)	5.6765 (4), 23.4382 (14), 6.9655 (6)
β (°)	104.620 (9)
*V* (Å^3^)	896.73 (12)
*Z*	4
Radiation type	Mo *K*α
μ (mm^−1^)	2.61
Crystal size (mm)	0.20 × 0.19 × 0.15

Data collection	
Diffractometer	Stoe *IPDS*-II
Absorption correction	Numerical (*X-RED* and *X-SHAPE*; Stoe, 2008 ▸)
*T*_min_, *T*_max_	0.470, 0.620
No. of measured, independent and observed [*I* > 2σ(*I*)] reflections	6594, 2163, 1704
*R*_int_	0.047
(sin θ/λ)_max_ (Å^−1^)	0.661

Refinement	
*R*[*F*^2^ > 2σ(*F*^2^)], *wR*(*F*^2^), *S*	0.036, 0.096, 1.03
No. of reflections	2163
No. of parameters	112
H-atom treatment	H-atom parameters constrained
Δρ_max_, Δρ_min_ (e Å^−3^)	0.49, −0.62

Computer programs: *X-AREA* (Stoe, 2008 ▸), *SHELXT* (Sheldrick, 2015*a* ▸), *SHELXL* (Sheldrick, 2015*b* ▸), *DIAMOND* (Brandenburg, 1999 ▸) and *XP* in *SHELXTL-PC* (Sheldrick, 2008 ▸) and *publCIF* (Westrip, 2010 ▸).

## supplementary crystallographic information

### Poly[(2,6-dimethylpyrazine-κN4)(µ3-thiocyanato-κ3N:S:S)copper(I)] Crystal data

**Table:**

[Cu(NCS)(C_6_H_8_N_2_)]	*F*(000) = 464
*M**_r_* = 229.76	*D*_x_ = 1.702 Mg m^−^^3^
Monoclinic, *P*2_1_/*c*	Mo *K*α radiation, λ = 0.71073 Å
*a* = 5.6765 (4) Å	Cell parameters from 5598 reflections
*b* = 23.4382 (14) Å	θ = 6.3–56.0°
*c* = 6.9655 (6) Å	µ = 2.61 mm^−^^1^
β = 104.620 (9)°	*T* = 200 K
*V* = 896.73 (12) Å^3^	Block, colorless
*Z* = 4	0.20 × 0.19 × 0.15 mm

### Poly[(2,6-dimethylpyrazine-κN4)(µ3-thiocyanato-κ3N:S:S)copper(I)] Data collection

**Table:**

Stoe IPDS-II diffractometer	1704 reflections with *I* > 2σ(*I*)
ω scans	*R*_int_ = 0.047
Absorption correction: numerical (X-Red and X-Shape; Stoe, 2008)	θ_max_ = 28.0°, θ_min_ = 3.2°
*T*_min_ = 0.470, *T*_max_ = 0.620	*h* = −7→7
6594 measured reflections	*k* = −29→30
2163 independent reflections	*l* = −9→9

### Poly[(2,6-dimethylpyrazine-κN4)(µ3-thiocyanato-κ3N:S:S)copper(I)] Refinement

**Table:**

Refinement on *F*^2^	Hydrogen site location: inferred from neighbouring sites
Least-squares matrix: full	H-atom parameters constrained
*R*[*F*^2^ > 2σ(*F*^2^)] = 0.036	*w* = 1/[σ^2^(*F*_o_^2^) + (0.0586*P*)^2^] where *P* = (*F*_o_^2^ + 2*F*_c_^2^)/3
*wR*(*F*^2^) = 0.096	(Δ/σ)_max_ = 0.001
*S* = 1.03	Δρ_max_ = 0.49 e Å^−^^3^
2163 reflections	Δρ_min_ = −0.62 e Å^−^^3^
112 parameters	Extinction correction: SHELXL-2016/6 (Sheldrick 2015b), Fc^*^=kFc[1+0.001xFc^2^λ^3^/sin(2θ)]^-1/4^
0 restraints	Extinction coefficient: 0.010 (3)

### Poly[(2,6-dimethylpyrazine-κN4)(µ3-thiocyanato-κ3N:S:S)copper(I)] Special details

**Table:**

Geometry. All esds (except the esd in the dihedral angle between two l.s. planes) are estimated using the full covariance matrix. The cell esds are taken into account individually in the estimation of esds in distances, angles and torsion angles; correlations between esds in cell parameters are only used when they are defined by crystal symmetry. An approximate (isotropic) treatment of cell esds is used for estimating esds involving l.s. planes.

### Poly[(2,6-dimethylpyrazine-κN4)(µ3-thiocyanato-κ3N:S:S)copper(I)] Fractional atomic coordinates and isotropic or equivalent isotropic displacement parameters (Å^2^)

**Table:**

	*x*	*y*	*z*	*U*_iso_*/*U*_eq_	
Cu1	0.69476 (6)	0.27873 (2)	0.52022 (5)	0.02275 (15)	
N1	0.7931 (5)	0.43852 (12)	0.1025 (4)	0.0296 (6)	
C1	0.5987 (6)	0.40441 (13)	0.0613 (4)	0.0281 (6)	
C2	0.5679 (6)	0.36229 (13)	0.1940 (4)	0.0252 (6)	
H2	0.423880	0.339862	0.163200	0.030*	
N2	0.7354 (4)	0.35266 (11)	0.3629 (3)	0.0226 (5)	
C3	0.9327 (5)	0.38613 (14)	0.4014 (4)	0.0259 (6)	
H3	1.057102	0.379901	0.519348	0.031*	
C4	0.9611 (6)	0.42982 (13)	0.2736 (4)	0.0253 (6)	
C5	0.4140 (8)	0.41223 (19)	−0.1321 (6)	0.0507 (11)	
H5A	0.396766	0.452951	−0.164523	0.076*	
H5B	0.256982	0.396923	−0.121909	0.076*	
H5C	0.467254	0.391925	−0.236799	0.076*	
C6	1.1765 (7)	0.46912 (17)	0.3216 (5)	0.0373 (8)	
H6A	1.226703	0.477786	0.200078	0.056*	
H6B	1.311239	0.450682	0.417527	0.056*	
H6C	1.132326	0.504568	0.378469	0.056*	
S1	0.88125 (12)	0.29030 (3)	0.85550 (10)	0.02064 (18)	
C11	1.1536 (5)	0.25913 (13)	0.9018 (4)	0.0187 (5)	
N11	1.3454 (4)	0.23893 (12)	0.9428 (3)	0.0243 (5)	

### Poly[(2,6-dimethylpyrazine-κN4)(µ3-thiocyanato-κ3N:S:S)copper(I)] Atomic displacement parameters (Å^2^)

**Table:**

	*U*^11^	*U*^22^	*U*^33^	*U*^12^	*U*^13^	*U*^23^
Cu1	0.01201 (19)	0.0303 (2)	0.0246 (2)	−0.00085 (13)	0.00210 (13)	0.00651 (13)
N1	0.0388 (15)	0.0256 (14)	0.0223 (12)	−0.0007 (11)	0.0039 (11)	0.0035 (9)
C1	0.0351 (16)	0.0231 (16)	0.0208 (13)	0.0000 (13)	−0.0028 (12)	0.0016 (11)
C2	0.0276 (14)	0.0204 (15)	0.0236 (13)	0.0012 (11)	−0.0012 (11)	0.0008 (11)
N2	0.0247 (12)	0.0197 (12)	0.0200 (11)	−0.0019 (9)	−0.0005 (9)	0.0043 (9)
C3	0.0243 (14)	0.0271 (16)	0.0218 (13)	−0.0043 (11)	−0.0023 (11)	0.0030 (10)
C4	0.0263 (15)	0.0246 (15)	0.0246 (14)	−0.0034 (12)	0.0053 (11)	−0.0017 (11)
C5	0.057 (2)	0.049 (2)	0.0310 (18)	−0.008 (2)	−0.0170 (17)	0.0144 (16)
C6	0.0353 (18)	0.038 (2)	0.0378 (18)	−0.0144 (15)	0.0071 (14)	0.0014 (13)
S1	0.0139 (3)	0.0280 (4)	0.0194 (3)	0.0041 (2)	0.0030 (2)	0.0007 (2)
C11	0.0148 (12)	0.0262 (14)	0.0146 (11)	−0.0048 (10)	0.0031 (9)	−0.0029 (9)
N11	0.0114 (10)	0.0392 (15)	0.0212 (11)	0.0020 (10)	0.0021 (8)	−0.0027 (10)

### Poly[(2,6-dimethylpyrazine-κN4)(µ3-thiocyanato-κ3N:S:S)copper(I)] Geometric parameters (Å, º)

**Table:**

Cu1—N11^i^	1.963 (2)	C3—C4	1.393 (4)
Cu1—N2	2.094 (2)	C3—H3	0.9500
Cu1—S1	2.3238 (8)	C4—C6	1.500 (4)
Cu1—S1^ii^	2.3809 (8)	C5—H5A	0.9800
N1—C1	1.334 (4)	C5—H5B	0.9800
N1—C4	1.341 (4)	C5—H5C	0.9800
C1—C2	1.393 (4)	C6—H6A	0.9800
C1—C5	1.495 (5)	C6—H6B	0.9800
C2—N2	1.332 (4)	C6—H6C	0.9800
C2—H2	0.9500	S1—C11	1.666 (3)
N2—C3	1.338 (4)	C11—N11	1.155 (4)
			
N11^i^—Cu1—N2	105.53 (10)	N1—C4—C6	117.5 (3)
N11^i^—Cu1—S1	118.22 (7)	C3—C4—C6	121.9 (3)
N2—Cu1—S1	110.30 (7)	C1—C5—H5A	109.5
N11^i^—Cu1—S1^ii^	105.40 (8)	C1—C5—H5B	109.5
N2—Cu1—S1^ii^	100.90 (7)	H5A—C5—H5B	109.5
S1—Cu1—S1^ii^	114.74 (3)	C1—C5—H5C	109.5
C1—N1—C4	117.7 (3)	H5A—C5—H5C	109.5
N1—C1—C2	121.2 (3)	H5B—C5—H5C	109.5
N1—C1—C5	118.3 (3)	C4—C6—H6A	109.5
C2—C1—C5	120.5 (3)	C4—C6—H6B	109.5
N2—C2—C1	121.7 (3)	H6A—C6—H6B	109.5
N2—C2—H2	119.2	C4—C6—H6C	109.5
C1—C2—H2	119.2	H6A—C6—H6C	109.5
C2—N2—C3	116.9 (3)	H6B—C6—H6C	109.5
C2—N2—Cu1	117.0 (2)	C11—S1—Cu1	107.96 (9)
C3—N2—Cu1	125.3 (2)	C11—S1—Cu1^iii^	96.46 (10)
N2—C3—C4	122.0 (3)	Cu1—S1—Cu1^iii^	105.06 (3)
N2—C3—H3	119.0	N11—C11—S1	176.6 (3)
C4—C3—H3	119.0	C11—N11—Cu1^iv^	168.0 (3)
N1—C4—C3	120.6 (3)		
			
C4—N1—C1—C2	1.8 (5)	C2—N2—C3—C4	1.2 (4)
C4—N1—C1—C5	−177.9 (3)	Cu1—N2—C3—C4	170.5 (2)
N1—C1—C2—N2	−2.9 (5)	C1—N1—C4—C3	0.7 (5)
C5—C1—C2—N2	176.7 (3)	C1—N1—C4—C6	−178.6 (3)
C1—C2—N2—C3	1.3 (4)	N2—C3—C4—N1	−2.3 (5)
C1—C2—N2—Cu1	−168.9 (2)	N2—C3—C4—C6	177.0 (3)

Symmetry codes: (i) *x*−1, −*y*+1/2, *z*−1/2; (ii) *x*, −*y*+1/2, *z*−1/2; (iii) *x*, −*y*+1/2, *z*+1/2; (iv) *x*+1, −*y*+1/2, *z*+1/2.
